# When the Aorta Deceives: A Type A Dissection Case Masquerading as Limb Ischemia and Fecal Incontinence

**DOI:** 10.7759/cureus.99968

**Published:** 2025-12-23

**Authors:** Ebtesam Safi, Mahgob Alobied, Lubna Saffarini, Maher Y AlObeid

**Affiliations:** 1 Emergency Medicine, Rashid Hospital, Dubai, ARE; 2 Institute of Learning, Master of Science in Health Professions Education (MScHPE), Mohammed Bin Rashid University of Medicine and Health Sciences, Dubai, ARE

**Keywords:** aortic dissection, atypical presentation, case report, emergency, stanford type a

## Abstract

A 55-year-old hypertensive male and chronic smoker presented with sudden severe central chest pain accompanied by excruciating left lower-limb pain and episodes of non-bloody diarrhea associated with fecal incontinence. Examination revealed a cold, clammy right upper limb and left lower limb with diminished peripheral pulses but no neurological deficits or abdominal tenderness. CT aortography demonstrated a Stanford Type A aortic dissection extending from the aortic annulus to the iliac arteries. The patient was stabilized and then transferred for emergent open surgical repair. Aortic dissection is an acute cardiovascular emergency characterized by an intimal tear in the aortic wall, allowing blood to dissect between the intima and media, compromising flow to vital organs. It is associated with exceedingly high morbidity and mortality rates. It is commonly associated with uncontrolled hypertension, connective tissue disorders, and atherosclerosis. This case highlights the importance of urgent management and early recognition of atypical manifestations, such as gastrointestinal symptoms and limb ischemia in acute aortic dissection, to enable rapid surgical intervention and favorable outcomes.

## Introduction

Acute aortic dissection is a life-threatening cardiovascular emergency with high mortality if left untreated. Stanford Type A dissections that involve the ascending aorta require immediate surgical management. Classic presentations include sudden, severe ‘’tearing’’ chest pain, but atypical features such as limb ischemia or gastrointestinal symptoms can obscure diagnosis and delay treatment. A significant proportion of aortic dissections go unrecognized on initial presentation to the emergency department, with only 15%-43% accurately diagnosed at first encounter; hence, proper head-to-toe examination is crucial [1]. We report a rare case of extensive Stanford Type A dissection with simultaneous upper and lower limb malperfusion as well as gastrointestinal symptoms.

## Case presentation

A 55-year-old man, known hypertensive for 33 years on amlodipine/valsartan 10 mg/160 mg once daily and a chronic smoker for 40 years, presented to the emergency department (ED) with the sudden onset of heavy central chest pain radiating to the throat. He rated the pain as 10/10. The pain began abruptly two hours prior to presentation and was described as unlike any previous pain. He reported severe pain in the left lower limb, as well as inability to move it and multiple episodes of non-bloody diarrhea with fecal incontinence that started the same day. There was no history of fever, trauma, abdominal pain, difficulty breathing, vomiting, or syncope.

On arrival, he was in severe distress and pain but hemodynamically stable with blood pressure (BP) of 152/54 mmHg, pulse of 65 beats per minute (bpm), respiration rate of 19, temperature of 36.5°C, and oxygen saturation of 98%.

For the primary assessment, the patient had a patent airway with no signs of respiratory distress. Circulation revealed a pale, cold, and clammy right upper limb with absent peripheral pulse and delayed capillary refill, while the left lower limb was similarly cold and clammy with a diminished pulse but no discoloration. Examination of the abdomen demonstrated a soft, non-tender abdomen without peritoneal signs. Disability assessment showed a Glasgow Coma Scale of 15/15 [2], with no focal neurological deficits and normal blood glucose of 122 mg/dL. Exposure was unremarkable. Due to the pulse deficit, blood pressure was measured in the opposite right arm and was found to be 59/35 mmHg. 

Table 1 depicts the initial relevant laboratory investigations. Full blood count showed an elevated white blood cell count of 14.1 × 10⁹/L. Inflammatory markers and troponin levels were within normal limits. Lactate was found to be 5.3 mmol/L. Renal function showed a creatinine of 1.56 mg/dL with an estimated glomerular filtration rate of 52.1 mL/min/1.73 m². Urea and electrolyte levels were within normal limits except for hypokalemia with a potassium level of 2.8 mmol/L. Coagulation profile demonstrated a D-dimer level >20 µg/mL, a prothrombin time of 16.8 seconds, and an international normalized ratio (INR) of 1.21. 

**Table 1:** Relevant initial laboratory investigations.

Test	Result	Reference range
White blood cell count	14.1	3.6-11.0 × 10^3^/uL
C-reactive protein	4	<5.0 mg/dL
Troponin	11	<14 ng/L
Lactate	5.3	0.5-2.3 mmol/L
Creatinine	1.56	0.70-1.20 mg/dL
Estimated glomerular filtration rate	52.1	>60 mL/min/1.73 m²
Potassium	2.8	3.4-4.5 mmol/L
D-Dimer	>20	<0.5 ug/ml
Prothrombin time	16.8	12.6-16.3 seconds
International normalized ratio	1.21	0.8-1.1

**Figure 1:**
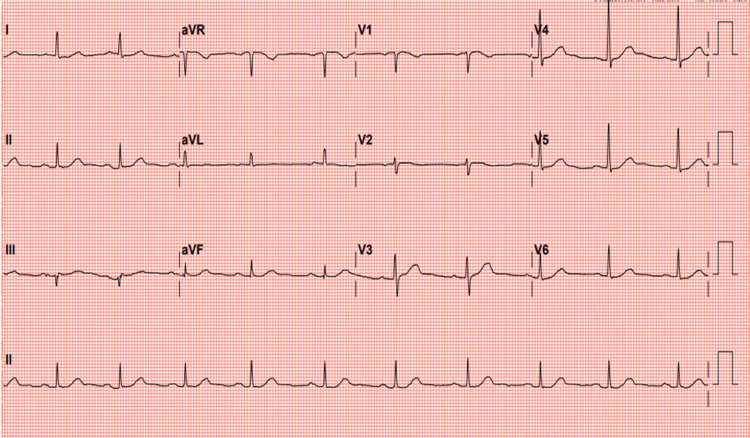
Electrocardiogram showing normal sinus rhythm with no evidence of any abnormalities.

**Figure 2:**
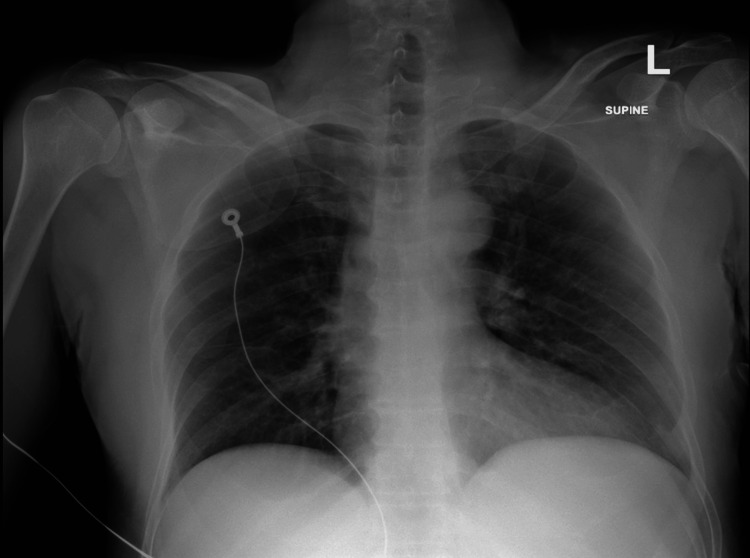
Chest radiograph demonstrating an ill-defined left retrocardiac opacity with associated aortic unfolding.

**Figure 3:**
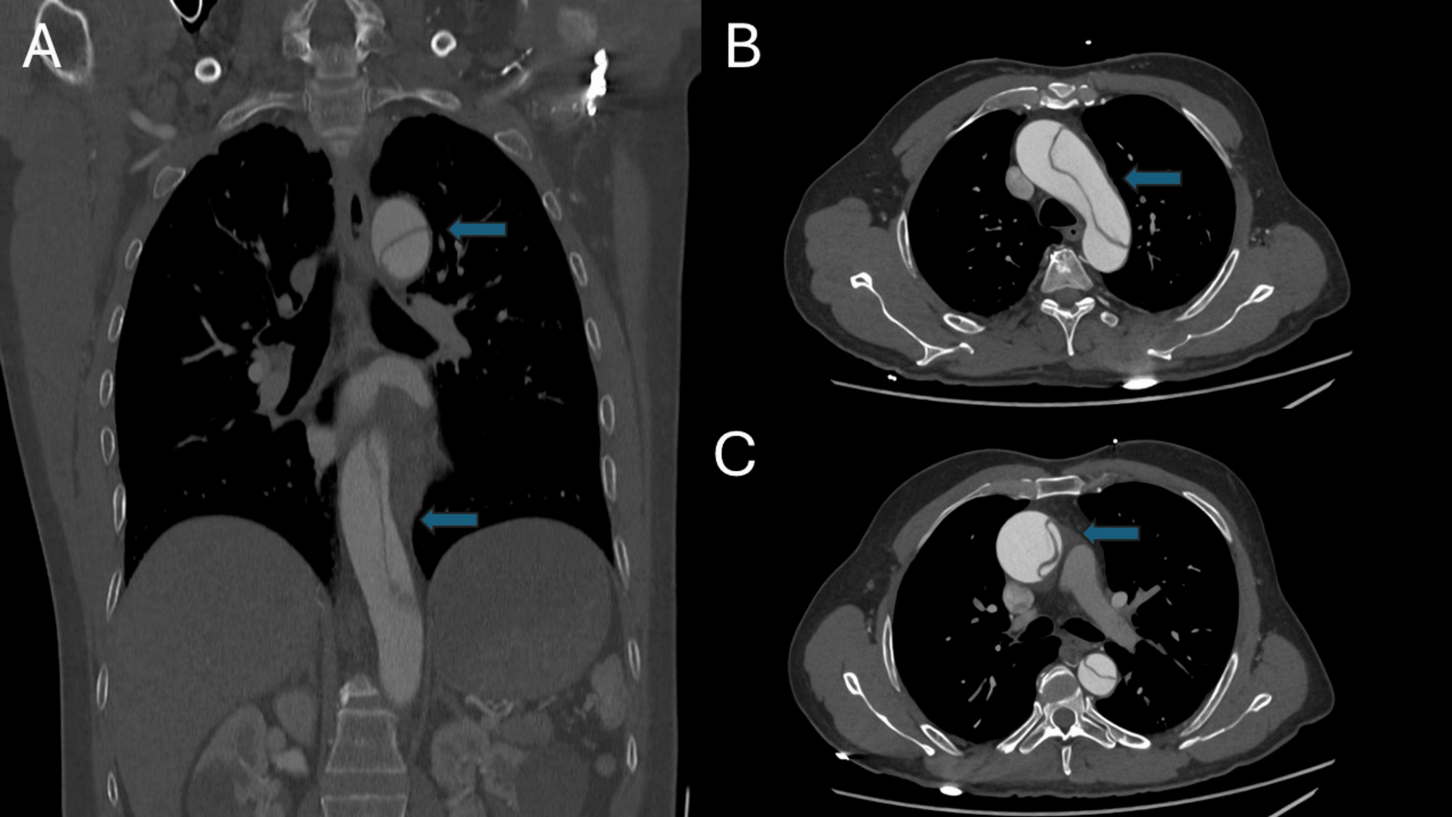
Multiple computed tomography angiography (CTA) scan slices showing extensive Stanford type A acute aortic dissection. The coronal image on the left (A) shows the extensive Stanford type A acute aortic dissection. Axial view (B) and (C) shows the intimal flaps. The intimal flap separates the true lumen (smaller caliber) from the false lumen (larger caliber) and is depicted by the blue arrows.

Point-of-care ultrasound (POCUS) was limited and suboptimal due to the patient’s body habitus, but the aortic diameter appeared normal with no flaps visualized, and no evidence of pulmonary embolism or intracardiac clots was noted. The electrocardiogram (Figure 1) demonstrates a normal sinus rhythm without ischemic changes or conduction blocks identified. Chest X-ray revealed an ill-defined opacity in the left retrocardiac region, suggestive of aspiration with unfolding of the aorta as seen in Figure 2. Transthoracic echocardiography demonstrated normal left ventricular size and preserved systolic function (ejection fraction 60-65%). Mild aortic regurgitation was noted, with no pericardial effusion. The ascending aorta was dilated (4.5 cm), with evidence of an aortic dissection extending from the aortic root near the sinotubular junction. A contrast-enhanced CT aortogram, however, confirmed a Stanford Type A aortic dissection as depicted in Figures 3A-3C below, originating at the aortic annulus and extending through the ascending aorta, aortic arch, and descending aorta down to the distal descending segment, involving the left external and internal iliac arteries.

Analgesia was provided with an initial 100 µg of intravenous (IV) dose of fentanyl followed by 10 mg of morphine, to which the patient responded well and his pain score was 3/10. Intravenous antihypertensives, including two 20 mg boluses of labetalol, were given to achieve and maintain a heart rate below 60 bpm and a systolic BP below 100-120 mmHg. He was then continued on an infusion of labetalol at a rate of 1-5 mg/min. The patient was managed with correction of hypokalemia using 40 mmol of potassium chloride administered over four hours via a peripheral line. The vascular and cardiothoracic surgical teams were urgently consulted, and the patient was transferred within the same organization for emergent life-saving surgical repair of the aortic dissection.

The patient underwent emergent replacement of the ascending aorta with a vascular graft, and the procedure was uneventful. He was admitted to the surgical intensive care unit for a total of 43 days, during which he developed acute kidney injury with oliguria, requiring continuous renal replacement therapy. His creatinine peaked at 3.2 mg/dL but improved to 1.02 mg/dL by discharge. He also had persistent *Enterobacter cloacae* bacteremia, which was treated with meropenem 2 g every eight hours for ten days. He was on deep vein thrombosis prophylaxis throughout. Difficult weaning from sedation and mechanical ventilation, likely due to suspected anoxic encephalopathy, necessitated tracheostomy on day 13. The patient was later discharged in stable condition and fit to travel to his home country for continuation of care.

## Discussion

Recent literature identifies acute aortic dissection as a rare but highly fatal cardiovascular event, with incidence rates estimated at four to seven per 100,000 population annually [3]. The majority of cases occur in individuals aged 50 to 70, with a mean age at presentation between 65 and 72 years [1]. Men are three times more likely to be affected than women, yet women often present at an older age and with worse outcomes [4]. Type A dissection, involving the ascending aorta, is more common and carries a significant mortality risk, with population-based studies reporting 30-day mortality rates of 28% for type A and 11% for type B dissections, highlighting the urgency required for diagnosis and intervention. Hypertension is present in up to 75% of cases and remains the most common modifiable risk factor [1].

Pathophysiologically, aortic dissection results from a tear in the intimal layer of the aorta, which allows blood to penetrate the medial layer, forming a false lumen that can extend along the vessel and disrupt blood supply. This process is driven by underlying conditions such as chronic hypertension, which induces degenerative changes as well as connective tissue disorders like Marfan syndrome and prior cardiac or aortic interventions. The propagation of the dissection and resulting compromise of aortic branches may rapidly lead to catastrophic sequelae, including rupture, cardiac tamponade, organ ischemia, and aortic valve regurgitation, all of which contribute to the high early mortality observed in acute cases [1,4].

Acute Stanford type A aortic dissection remains a life-threatening emergency requiring rapid recognition and management, particularly in the emergency department. Most patients, including cases like the 55-year-old hypertensive chronic smoker described here, present with abrupt severe chest pain but may also manifest atypical symptoms such as limb ischemia, pulse deficits, and gastrointestinal complaints, including diarrhea or fecal incontinence, emphasizing the importance of caution for non-classical features. These non-classical manifestations are rarely reported in the literature yet carry a substantial risk of missed or delayed diagnosis with potentially fatal consequences and increased mortality [5-7].

Recent literature highlights that more than half of patients with acute aortic dissection have atypical presentations, often normal vital signs and subtle physical findings. Emergency physicians must maintain a high index of suspicion, especially in older, hypertensive, or smoking patients presenting with sudden pain, ischemic limb features, or gastrointestinal symptoms [7,8]. Clinical assessment should focus on rapid identification of chest or abdominal pain, pulse deficits, neurologic symptoms, and evidence of branch-vessel malperfusion [8]. High-quality CT angiography is the imaging modality of choice and gold standard, while bedside transesophageal echocardiography remains valuable in unstable cases. It’s rapid, reliable, and considered to have comparable diagnostic performance. Additionally, bedside point-of-care ultrasound and transthoracic echocardiography may also aid in the diagnosis, particularly in hemodynamically unstable patients [9].

Initial ED management prioritizes strict hemodynamic stabilization. Evidence supports targeting systolic blood pressure between 100 and 120 mmHg and heart rate below 60 bpm, using intravenous beta-blockers (esmolol, labetalol) as first-line agents. Vasodilators (nicardipine, nitroprusside) may be added for resistant hypertension once beta-blockade is achieved [1,8,9]. Rapid pain control, preferably with morphine, helps decrease sympathetic tone and further lowers aortic shear stress [1,5]. Volume resuscitation and vasopressors should be reserved for hypotensive patients, with careful monitoring to avoid exacerbating the dissection [1]. Time to hemodynamic target is critical, with recommendations to reach safe BP and HR within 20-30 minutes of ED arrival [10].

Surgical consultation must be immediate, as definitive management of type A dissection is emergent open repair using synthetic grafts. While rare case reports describe conservative therapy and novel pharmacologic adjuncts such as tranexamic acid in select situations, surgery is the standard of care for nearly all patients [8,9]. Notably, multi-organ malperfusion, limb, gut, or renal involvement correlates with increased mortality and requires transfer to a cardiothoracic surgical center [11].

Several recent case reports reiterate that delayed or missed diagnosis, especially due to atypical presentations, substantially worsens outcomes, reinforcing the necessity for thorough re-evaluation and multidisciplinary team activation. Continuous monitoring, patient reassessment, and use of standardized pathways have demonstrated benefit in improving both diagnostic rates and in-hospital mortality [6,7].

In summary, emergency physicians play an essential role in early detection, targeted blood pressure, and heart rate control as per current guidelines, and coordination of advanced surgical management for acute type A aortic dissection. This case emphasizes the importance of maintaining a high index of suspicion for aortic dissection in patients with severe chest pain and atypical systemic findings, ensuring urgent imaging and multidisciplinary management.

## Conclusions

This case highlights that extensive Stanford Type A aortic dissection can present with atypical features, including gastrointestinal symptoms such as diarrhea and fecal incontinence, as well as limb ischemia, which may obscure the classic presentation. Such atypical presentations are exceedingly rare but are associated with significant diagnostic delay and increased mortality. Simultaneous malperfusion of limbs should raise suspicion for branch-vessel involvement and prompt urgent imaging. Early recognition, rapid blood pressure and heart rate control, adequate analgesia, and emergent surgical intervention through multidisciplinary coordination are critical to improving survival. Furthermore, modifiable risk factors such as hypertension and smoking remain central to the development and progression of aortic disease, emphasizing the importance of aggressive risk-factor management to prevent recurrence.
